# Audiometric Phenotypes of Noise-Induced Hearing Loss by Data-Driven Cluster Analysis and Their Relevant Characteristics

**DOI:** 10.3389/fmed.2021.662045

**Published:** 2021-03-25

**Authors:** Qixuan Wang, Minfei Qian, Lu Yang, Junbo Shi, Yingying Hong, Kun Han, Chen Li, James Lin, Zhiwu Huang, Hao Wu

**Affiliations:** ^1^Department of Otolaryngology-Head and Neck Surgery, Ninth People's Hospital, Shanghai Jiao Tong University School of Medicine, Shanghai, China; ^2^Ear Institute, Shanghai Jiao Tong University School of Medicine, Shanghai, China; ^3^Shanghai Key Laboratory of Translational Medicine on Ear and Nose Diseases, Shanghai, China; ^4^Hearing and Speech Center, Ninth People's Hospital, Shanghai Jiao Tong University School of Medicine, Shanghai, China; ^5^Network and Information Center, Shanghai Jiao Tong University, Shanghai, China

**Keywords:** noise-induced hearing loss, audiometric phenotype, notched audiogram, unsupervised learning, data-driven cluster analysis, multivariate characteristics

## Abstract

**Background:** The definition of notched audiogram for noise-induced hearing loss (NIHL) is presently based on clinical experience, but audiometric phenotypes of NIHL are highly heterogeneous. The data-driven clustering of subtypes could provide refined characteristics of NIHL, and help identify individuals with typical NIHL at diagnosis.

**Methods:** This cross-sectional study initially recruited 12,218 occupational noise-exposed employees aged 18–60 years from two factories of a shipyard in Eastern China. Of these, 10,307 subjects with no history of otological injurie or disease, family history of hearing loss, or history of ototoxic drug use were eventually enrolled. All these subjects completed health behavior questionnaires, cumulative noise exposure (CNE) measurement, and pure-tone audiometry. We did data-driven cluster analysis (k-means clustering) in subjects with hearing loss audiograms (*n* = 6,599) consist of two independent datasets (*n* = 4,461 and *n* = 2,138). Multinomial logistic regression was performed to analyze the relevant characteristics of subjects with different audiometric phenotypes compared to those subjects with normal hearing audiograms (*n* = 3,708).

**Results:** A total of 10,307 subjects (9,165 males [88.9%], mean age 34.5 [8.8] years, mean CNE 91.2 [22.7] dB[A]) were included, 3,708 (36.0%) of them had completely normal hearing, the other 6,599 (64.0%) with hearing loss audiograms were clustered into four audiometric phenotypes, which were replicable in two distinct datasets. We named the four clusters as the 4–6 kHz sharp-notched, 4–6 kHz flat-notched, 3–8 kHz notched, and 1–8 kHz notched audiogram. Among them, except for the 4–6 kHz flat-notched audiogram which was not significantly related to NIHL, the other three phenotypes with different relevant characteristics were strongly associated with noise exposure. In particular, the 4–6 kHz sharp-notched audiogram might be a typical subtype of NIHL.

**Conclusions:** By data-driven cluster analysis of the large-scale noise-exposed population, we identified three audiometric phenotypes associated with distinct NIHL subtypes. Data-driven sub-stratification of audiograms might eventually contribute to the precise diagnosis and treatment of NIHL.

## Introduction

Noise-induced hearing loss (NIHL) is one of the most common hearing loss in adults (1), with increasing incidence in children and adolescents (2) due to widespread recreational and transport noise exposure (3, 4). The World Health Organization (WHO) estimates that 10% of the world population is exposed to sound levels that could potentially cause NIHL (5). To date, treatment options for NIHL are limited, while ~50% of this burden could be prevented by early detection of NIHL, avoidance of noise exposure, prompt intervention, etc. (6).

It is widely accepted that the noise exposure usually causes high-frequency sensorineural hearing impairment (7, 8). Despite several previously concluded abstract phenotypes of NIHL including the high-frequency audiometric notch and the bulge downwards audiogram (9), there are still no clear audiometric criteria on stratifications of NIHL, which makes it difficult to specifically evaluate NIHL during clinical and primary health care (10, 11). One reason for this is the heterogeneous audiometric phenotypes of NIHL, involving complex confounding influencing factors. The majority of studies have adopted different definitions of high-frequency hearing loss (12, 13) and notched audiogram (14–16), which were chosen mainly by specialized intuition or clinical experience, rather than by data-driven analysis. These inconsistent assessment methods were manifested by various ranges of frequency and degrees of hearing loss, which may represent different subtypes of NIHL with inconsistent responses to intervention, and inevitably result in incomparable conclusions between studies.

Generally, descriptions of NIHL phenotypes are limited by subjectivity and poor data support. A data-driven classification that incorporates the multifrequency audiogram of NIHL is needed to identify subtypes with consistent patterns and characteristics. Cluster analysis is an unsupervised exploratory data mining technique able to group the most similar individuals with multiple specified variables in the same group called “cluster” without any previously defined hypothesis (17). Since audiogram stratification is based on the complex non-linear combination of thresholds at several frequencies, unbiased data-driven cluster analysis has recently been found to be a useful method for the identification of audiometric phenotypes (18, 19). We postulated that cluster analysis could be applied for classifying audiograms of NIHL.

In the current study, based on audiograms of 10,307 Chinese shipyard employees with various noise exposure levels, we used the k-means clustering algorithm to classify subtypes of NIHL in two distinct noise-exposed populations from different factories. The confounding influencing factors related to these subtypes were further analyzed to optimize the assessment for different subtypes of NIHL, which could provide a powerful tool to identify those individuals at great risk of NIHL and guide optimal prevention of noise exposure.

## Methods

### Study Population

**Figure 1:**
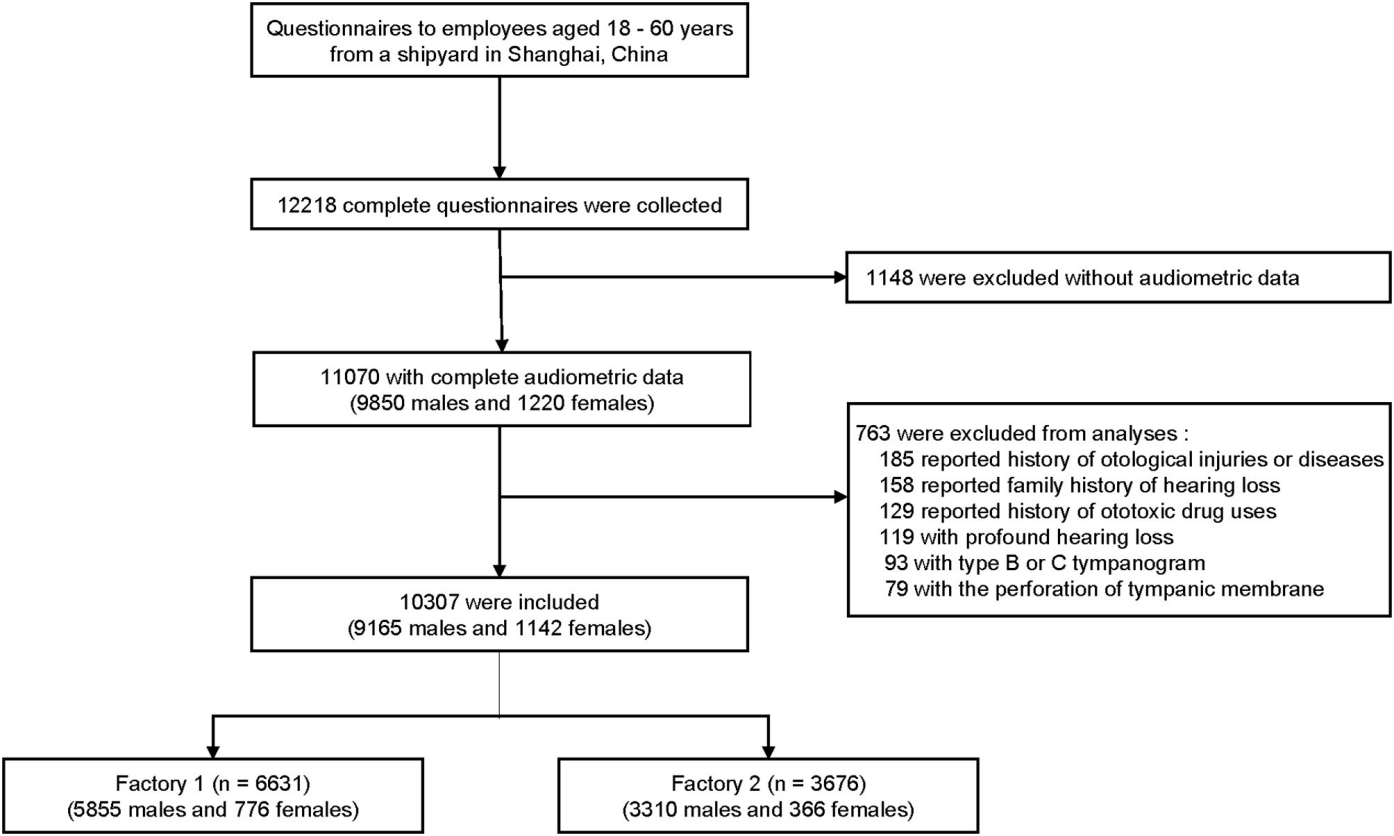
Flowchart of this cross-sectional study.

We conducted this hearing and health investigation in a shipyard in eastern China from August 1, 2017, to June 30, 2018. A total of 12,218 subjects aged 18–60 years were initially recruited, and 10,307 from two steel factories (6,631 from factory 1, and 3,676 from factory 2) were included in the analysis based on the following criteria: (1) completed questionnaire and audiometric data, (2) no history of otological injuries or diseases, (3) no family history of hearing loss, (4) no history of ototoxic drug use, (5) no profound hearing loss (average threshold at 0.5–2 kHz frequencies >70 dB HL in any ear), and (6) no perforation of tympanic membrane or abnormal tympanogram. Sex and race were self-reported. Figure 1 shows the flowchart of this cross-sectional study, which was in accordance with the Strengthening the Reporting of Observational Studies in Epidemiology (STROBE) reporting guidelines and approved by the ethics committee of the Ninth People's Hospital affiliated to Shanghai Jiao Tong University School of Medicine. All the participants signed written informed consent forms.

### Audiometry

Pure-tone air-conduction audiometry at frequencies of 0.5, 1, 2, 3, 4, 6, and 8 kHz in both ears was performed by certified audiological technicians using an audiometer (Otometrics Madsen, Xeta, Denmark) with TDH-39P headsets in a soundproof booth in accordance with the regulations of ISO 8253-1: 2010. The subjects were not exposed to occupational noise or loud sounds within 16 h before being examined. The average threshold of the left and right ears at each frequency was calculated for subsequent analysis without the age-correction according to ISO 7029: 2017, in order to avoid the artificial modification on the subsequent cluster analysis. Normal hearing was defined as hearing threshold ≤25 dB HL over 0.5–8 kHz frequencies. Hearing loss was defined as hearing threshold >25 dB HL at any frequency.

### Questionnaire

Demographic variables (sex, age, race, job type, working time-length) and behavioral characteristics, including hearing protection device (HPD) use (<4 h/work-day, ≥4 h/work-day), personal earphone use (<1 h/day, ≥1 h/day), tobacco (<10 cigarettes/day, ≥10 cigarettes/day) and alcohol (<50 g/day, ≥50 g/day) consumption, and auditory-related symptoms (hearing difficulty and tinnitus), were collected through a self-reported questionnaire. Body mass index (BMI) was measured and calculated by investigators, and then categorized into non-obese (<28 kg/m^2^) and obese (≥28 kg/m^2^) groups.

### Noise Exposure Dose

A composite quantitative noise exposure index, the cumulative noise exposure (CNE), was used to estimate the noise exposure level for each subject, which was calculated using the following formula (20):

$$\begin{array}{l} \text{CNE}=L_{\text{Aeq},8\text{h}}+10\text{log}T, \end{array}$$

where *L*_Aeq,8h_ is the equivalent sound pressure level in A weight of 8 continuous hours of a work-day, which was measured and analyzed using the personal exposure dosimeter (Aihua, ASV5910 type, Hangzhou, China). Subjects were required to wear the dosimeter on the shoulder for five work-days to calculate the average *L*_Aeq,8h_. *T* is the working time-length in years obtained from the questionnaire.

### Data-Driven Cluster Analysis

**Figure 2:**
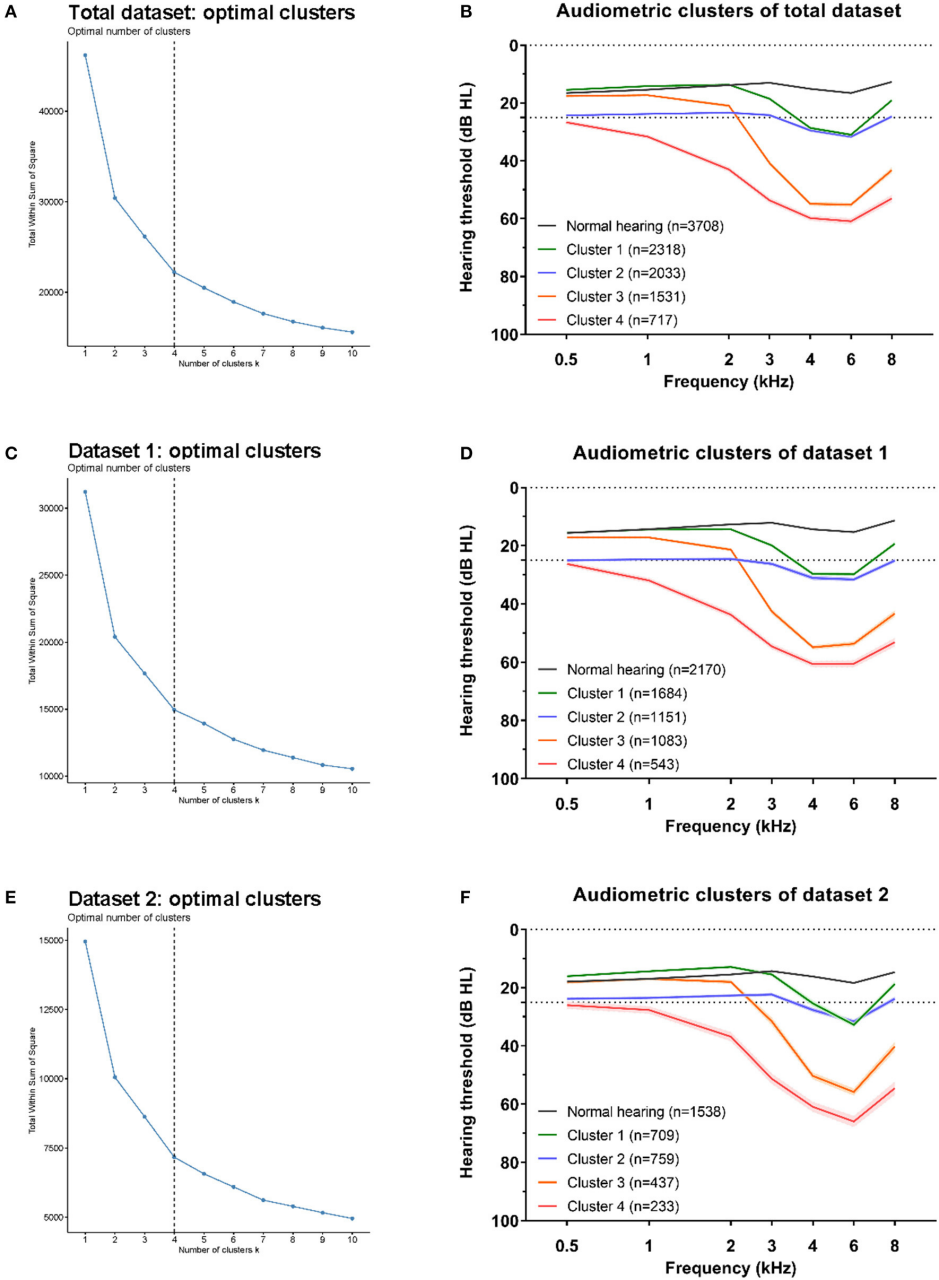
Optimal clusters for three datasets. The within cluster sum of squares (WSS) decrease with the increment of clusters number, and the optimal number of clustering was selected at the last one significantly reduced the WSS (at the inflection point of the curve). The optimal clustering were all at number of four (black dotted line) for total dataset **(A)**, dataset 1 **(C)**, and dataset 2 **(E)**. The average hearing thresholds over 0.5–8 kHz frequencies of normal hearing subjects and those four clusters were shown for total dataset **(B)**, dataset 1 **(D)**, and dataset 2 **(F)**.

Seven variables including standardized values of thresholds at frequencies of 0.5, 1, 2, 3, 4, 6, and 8 kHz were input for k-means cluster analysis performed using R software (version 4.0.3) (21). The optimal number of clusters was selected according to the within cluster sum of squares (WSS) (22), the number of clusters from 2 to 15 was tried, and the last one that significantly reduced the WSS (at the inflection point of the curve) was selected as the optimal number of clusters (Figures 2A–E). Data-driven cluster analysis was performed in data from two factories (dataset 1 and dataset 2) separately, and then repeated in the total data.

### Statistical Analysis

Data analysis was performed by using IBM SPSS version 24.0 software (SPSS Inc., Chicago, IL, USA) except for cluster analysis. Continuous variables are expressed as the mean (standard deviation, SD), and categorical variables are presented as percentages (*n* [%]). Statistical significance for differences in continuous variables was examined using Student's *t* test (between dataset 1 and dataset 2) or ANOVA (between cluster subtypes with Dunn-Bonferroni tests for *post-hoc* analyses), and categorical variables were compared by the chi-square test. Multinomial logistic regression models were used to analyze relevant factors of different clusters of audiometric subtypes. For the hierarchical regression, age was categorized into 3 groups (<30, 30–45, and >45 years). For all models, odds ratios (ORs) and 95% confidence intervals (CIs) are presented. A 2-tailed *P* < 0.05 was considered statistically significant.

## Results

### Basic Characteristics of Subjects

A total of 10,307 Chinese Han subjects (9,165 males [88.9%], mean age 34.5 [SD 8.8] years, mean CNE 91.2 [SD 22.7] dB[A]) were included. Among all subjects, 3,708 (36.0%) had completely normal hearing over 0.5–8 kHz frequencies. The total subjects were recruited from two independent factories in a shipyard, who had similar types of occupational tasks, despite significantly different distributions of sex, age, CNE, hearing loss, and other characteristics. The distributions of age, CNE, sex, BMI, hearing difficulty, tinnitus, HPD use, earphone use, tobacco consumption, and alcohol consumption are shown in Table 1.

**Table 1:** Characteristics of subjects in different datasets.

**Variables**	**Total (*n* = 10,307)**	**Dataset 1 (*n* = 6,631)**	**Dataset 2 (*n* = 3,676)**	*P* value^*^
Age (years), mean (SD)	34.5 (8.8)	36.2 (8.6)	31.4 (8.3)	<0.001
CNE (dB[A]), mean (SD)	91.2 (22.7)	92.0 (22.0)	89.8 (23.9)	<0.001
Sex, *n* (%)				0.007
Males	9,165 (88.9)	5,855 (88.3)	3,310 (90.0)	
Females	1,142 (11.1)	776 (11.7)	366 (10.0)	
BMI, *n* (%)				<0.001
Non-obese	9,391 (90.9)	6,000 (90.5)	3,371 (91.7)	
Obese	936 (9.1)	631 (9.5)	305 (8.3)	
Hearing difficulty, *n* (%)				<0.001
No	7,955 (77.2)	4,952 (74.7)	3,003 (81.7)	
Yes	2,352 (22.8)	1,679 (25.3)	673 (18.3)	
Tinnitus, *n* (%)				<0.001
No	6,971 (67.6)	4,327 (65.3)	2,644 (71.9)	
Yes	3,336 (32.4)	2,304 (34.7)	1,032 (28.1)	
HPD use, *n* (%)				<0.001
<4 h/work-day	7,384 (71.6)	4,936 (74.4)	2,448 (66.6)	
≥4 h/work-day	2,923 (28.4)	1,695 (25.6)	1,228 (33.4)	
Earphone use, *n* (%)				<0.001
<1 h/day	5,844 (56.7)	3,418 (51.5)	2,426 (66.0)	
≥1 h/day	4,463 (43.3)	3,213 (48.5)	1,250 (34.0)	
Tobacco consumption, *n* (%)				0.156
<10 cigarettes/day	6,436 (62.4)	4,174 (62.9)	2,262 (61.5)	
≥10 cigarettes/day	3,871 (37.6)	2,457 (37.1)	1,414 (38.5)	
Alcohol consumption, *n* (%)				<0.001
<50 g/day	7,558 (73.3)	5,076 (76.5)	2,482 (67.5)	
≥50 g/day	2,749 (26.7)	1,555 (23.5)	1,194 (32.5)	
Hearing loss, *n* (%)				<0.001
No	3,708 (36.0)	2,170 (32.7)	1,538 (41.8)	
Yes	6,599 (64.0)	4,461 (67.3)	2,138 (58.2)	

*SD, standard deviation; BMI, body mass index; CNE, cumulative noise exposure; HPD, hearing protective device*.

* *Comparisons were between dataset 1 and dataset 2*.

### Clusters of Audiometric Phenotypes

To classify NIHL into novel audiometric phenotypes, we used the k-means clustering method in audiograms with hearing loss. We repeated the cluster process, respectively, in total dataset (all the hearing loss audiograms, *n* = 6,599), dataset 1 (hearing loss audiograms from factory 1, *n* = 4,461) and dataset 2 (hearing loss audiograms from factory 2, *n* = 2,138) to verify that the cluster structure described for each dataset was reproducible.

For all three datasets, the optimal number of clusters was four according to the WSS decreasing curve (Figures 2A–E), and the audiometric phenotypes of four clusters identified from different datasets were qualitatively similar. In total, 6,239 /6,599 (94.5%) audiograms in total dataset clusters were classified into the same subtype according to the distinct clusters in dataset 1 (4,286 /4,461, 96.1%) and dataset 2 (1,953 /2,138, 91.3%), the consistency of subtypes by cluster analysis in two distinct datasets and total dataset showed in Table 2. The average hearing thresholds of normal hearing subjects and those four clusters are shown for each dataset (Figures 2B–F).

**Table 2:** The consistency of subtypes by cluster analysis in two independent datasets and total dataset.

**Consistency, *n* (%)**	**Cluster 1**	**Cluster 2**	**Cluster 3**	**Cluster 4**	**Total**
Dataset 1	1,549 (100.0)	1,123 (89.9)	1,075 (97.3)	539 (96.6)	4,286 (96.1)
Dataset 2	703 (91.4)	737 (94.0)	354 (83.1)	159 (100.0)	1,953 (91.3)
Total	2,252 (97.2)	1,860 (91.5)	1,492 (93.3)	698 (97.4)	6,239 (94.5)

### Relevant Characteristics of Audiometric Phenotypes

**Figure 3:**
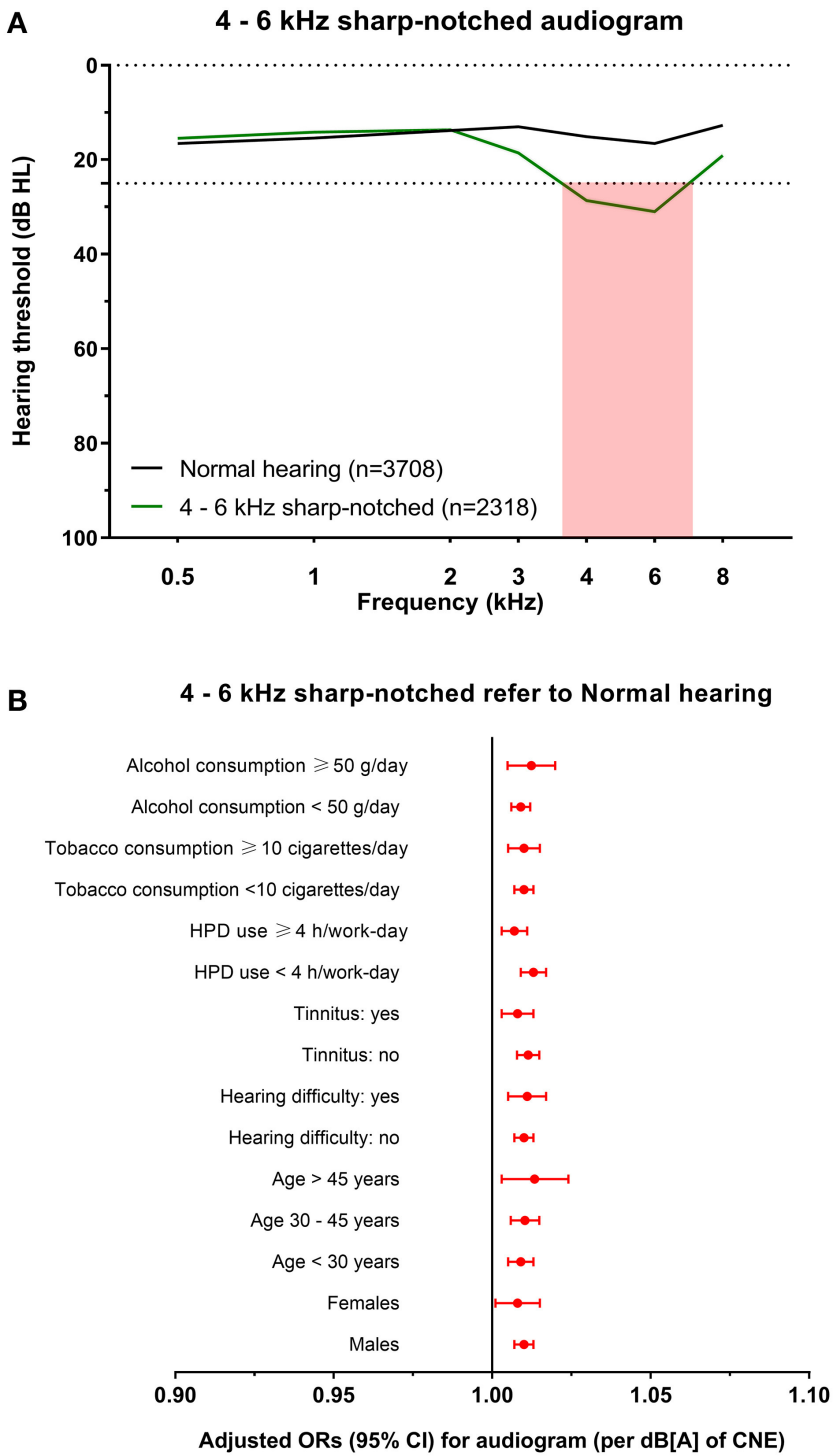
The 4–6 kHz sharp-notched audiogram and its association with noise exposure. **(A)** The average hearing thresholds over 0.5–8 kHz frequencies of subjects with normal hearing and the cluster of 4–6 kHz sharp-notched audiogram. The pink shade includes the range of notched frequencies. **(B)** Adjusted ORs with 95% CI of CNE increment (per dB[A]) for the 4–6 kHz sharp-notched audiogram refer to normal hearing audiogram after stratification of sex, age, hearing difficulty, tinnitus, HPD use, tobacco consumption, and alcohol consumption.

**Figure 4:**
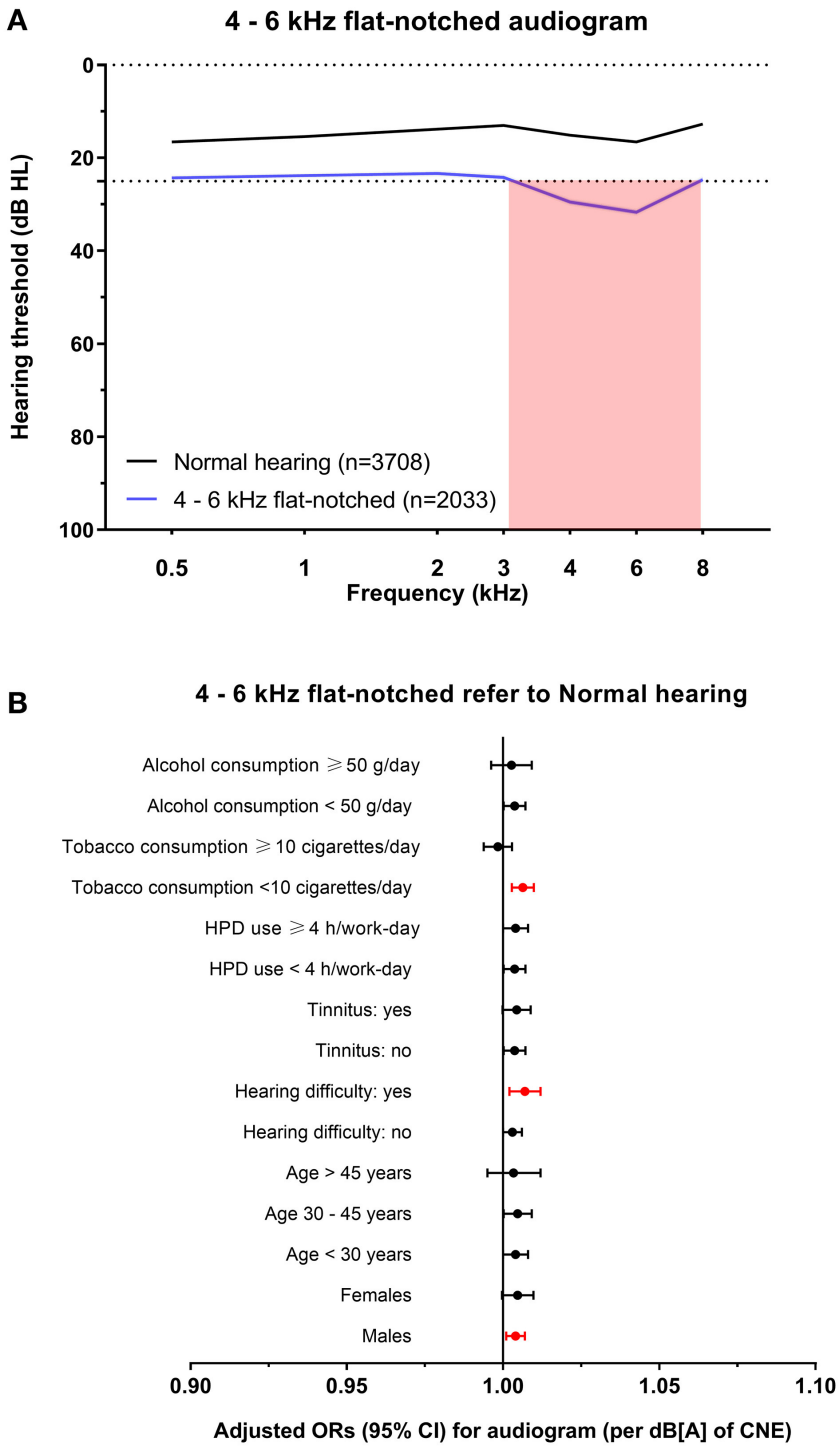
The 4–6 kHz flat-notched audiogram and its association with noise exposure. **(A)** The average hearing thresholds over 0.5–8 kHz frequencies of subjects with normal hearing and the cluster of 4–6 kHz flat-notched audiogram. The pink shade includes the range of notched frequencies. **(B)** Adjusted ORs with 95% CI of CNE increment (per dB[A]) for the 4–6 kHz flat-notched audiogram refer to normal hearing audiogram after stratification of sex, age, hearing difficulty, tinnitus, HPD use, tobacco consumption, and alcohol consumption.

**Figure 5:**
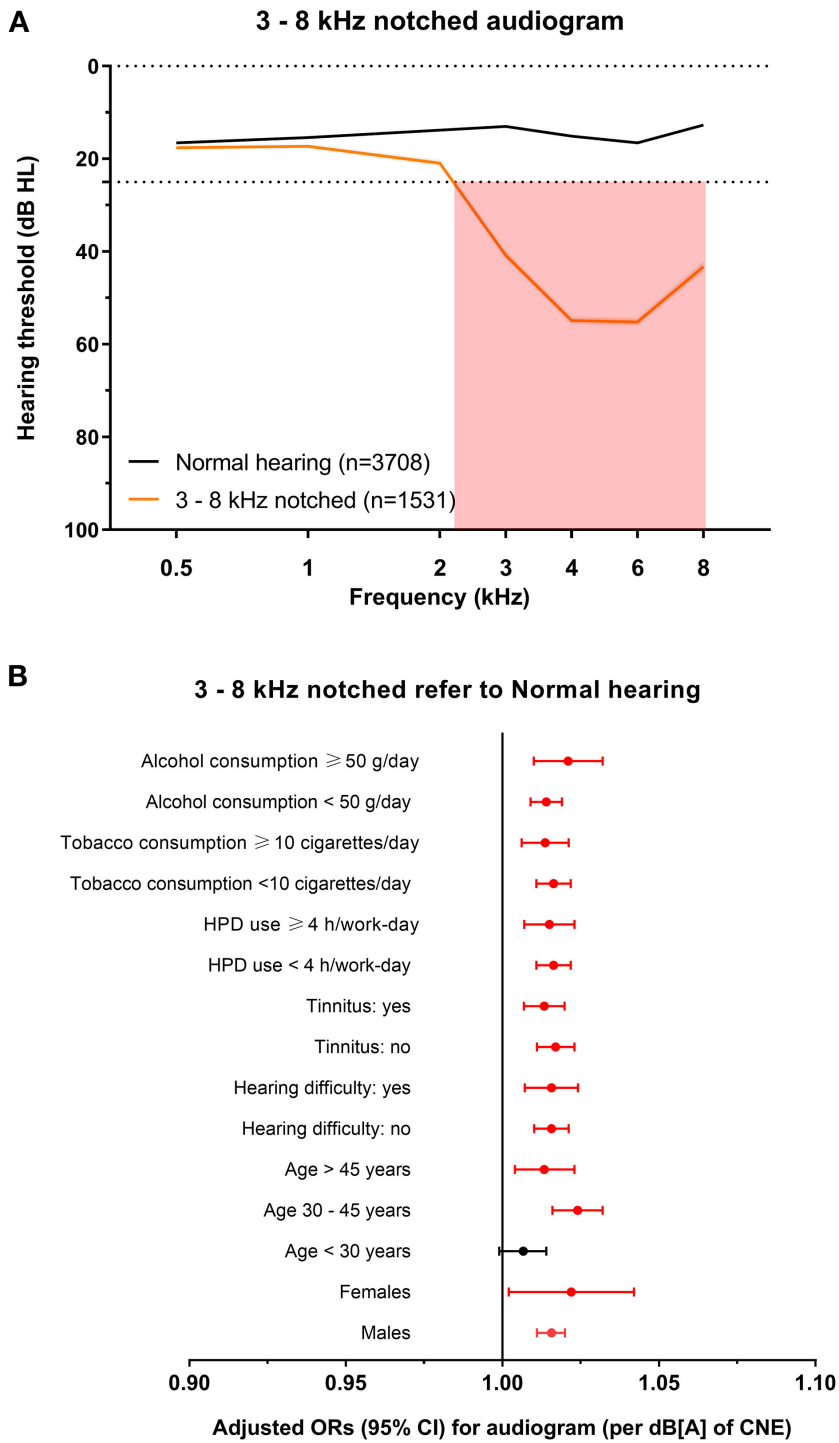
The 3–8 kHz notched audiogram and its association with noise exposure. **(A)** The average hearing thresholds over 0.5–8 kHz frequencies of subjects with normal hearing and the cluster of 3–8 kHz notched audiogram. The pink shade includes the range of notched frequencies. **(B)** Adjusted ORs with 95% CI of CNE increment (per dB[A]) for the 3–8 kHz notched audiogram refer to normal hearing audiogram after stratification of sex, age, hearing difficulty, tinnitus, HPD use, tobacco consumption, and alcohol consumption.

**Figure 6:**
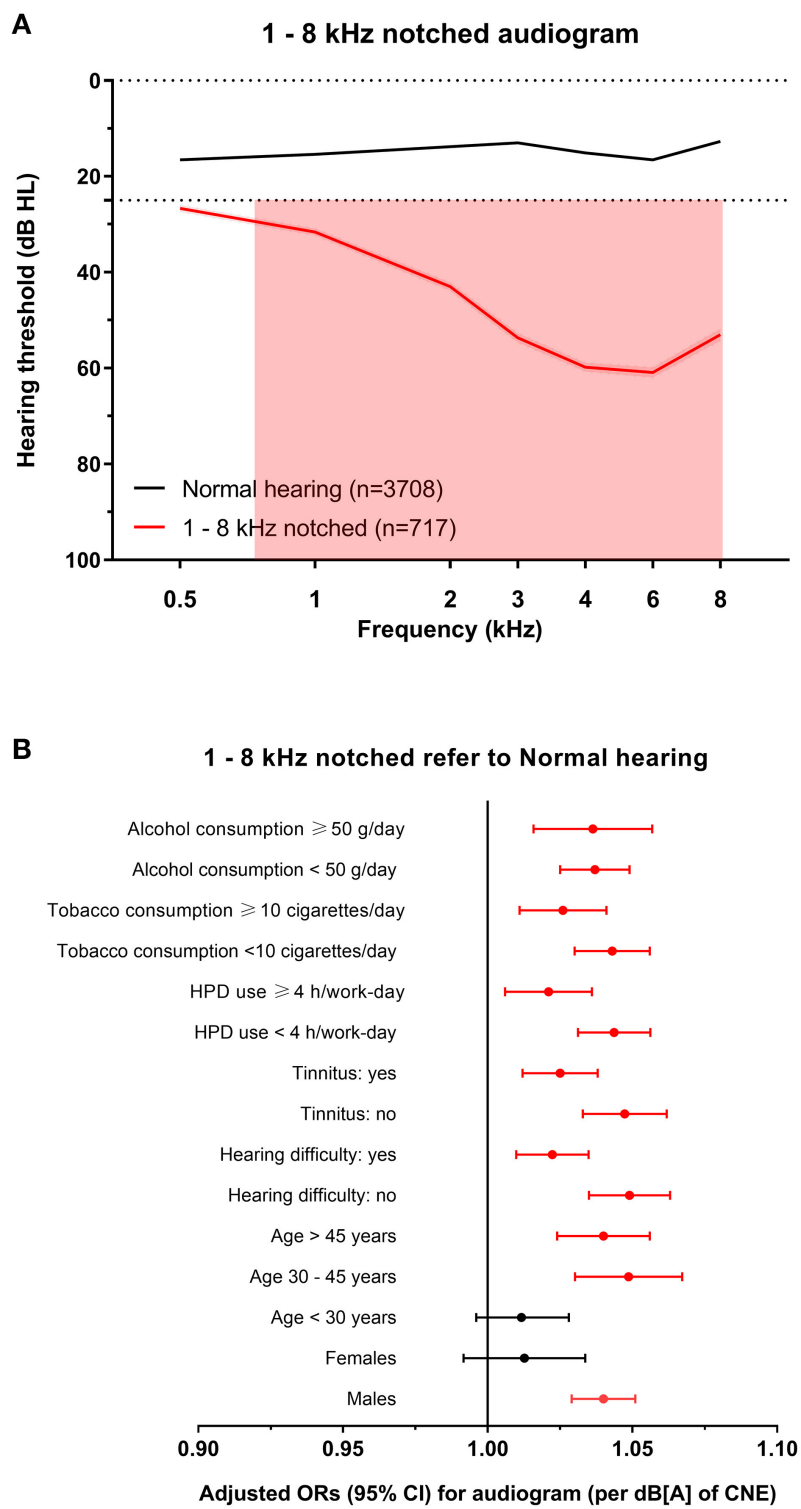
The 1–8 kHz notched audiogram and its association with noise exposure. **(A)** The average hearing thresholds over 0.5–8 kHz frequencies of subjects with normal hearing and the cluster of 1–8 kHz notched audiogram. The pink shade includes the range of notched frequencies. **(B)** Adjusted ORs with 95% CI of CNE increment (per dB[A]) for the 1–8 kHz notched audiogram refer to normal hearing audiogram after stratification of sex, age, hearing difficulty, tinnitus, HPD use, tobacco consumption, and alcohol consumption.

Audiograms with hearing loss were then classified into 4 subtypes for cluster analysis of the total dataset, which were named 4–6 kHz sharp-notched (original cluster 1, Figure 3A), 4–6 kHz flat-notched (original cluster 2, Figure 4A), 3–8 kHz notched (original cluster 3, Figure 5A), and 1–8 kHz notched (original cluster 4, Figure 6A) phenotypes, referring to the frequency range, and shape of their audiometric notches. Hearing thresholds at frequencies of 0.5–8 kHz of the four subtypes were significantly different from each other (all the *P* values < 0.001). In comparison with the 4–6 kHz sharp- and flat-notched subtypes, subjects manifested as the 3–8 kHz and 1–8 kHz notched subtypes were significantly older, with higher noise exposure, as well as higher proportions of males, hearing difficulties and tinnitus. In *post-hoc* analyses, for the 4–6 kHz flat-notched audiogram, the average age of subjects of this subtype was similar (*P* = 0.293) to that of the 4–6 kHz sharp-notched audiogram, while the mean CNE was slightly smaller (*P* = 0.008) than that of the 4–6 kHz sharp-notched audiogram, but significantly larger (*P* < 0.001) than that of the normal-hearing audiogram. The proportions of females, hearing difficulties, tinnitus, and earphone uses were higher in subjects with the 4–6 kHz flat-notched audiogram than that in the 4–6 kHz sharp-notched audiogram. Moreover, the average hearing thresholds of the 4–6 kHz flat-notched audiogram at frequencies of 0.5–3 kHz were obviously higher than that of the 4–6 kHz sharp-notched audiogram (all the *P* value < 0.001). The detailed distribution of characteristics in subjects with different audiometric phenotypes is shown in Table 3.

**Table 3:** Characteristics of subjects in different audiometric phenotypes.

**Variables**	**Normal hearing (*n* = 3,708)**	**4–6 kHz sharp-notched (*n* = 2,318)**	**4–6 kHz flat-notched (*n* = 2,033)**	**3–8 kHz notched (*n* = 1,531)**	**1–8 kHz notched (*n* = 717)**	*P* value^*^
Age (years), mean (SD)	29.9 (7.0)^b,c,d,e^	34.9 (7.7)^a,d,e^	35.4 (8.9)^a,d,e^	39.6 (8.1)^a,b,c,e^	43.5.4 (7.8)^a,b,c,d^	<0.001
CNE (dB[A]), mean (SD)	85.4 (27.8)^b,c,d,e^	93.8 (17.6)^a,c,d,e^	91.5 (22.6)^a,b,d,e^	96.9 (14.3)^a,b,c,e^	100.0 (13.3)^a,b,c,d^	<0.001
Sex, *n* (%)						<0.001
Males	3,065 (82.7)^b,c,d,e^	2,144 (92.5)^a,c,d,e^	1,775 (87.3)^a,b,d,e^	1,492 (97.5)^a,b,c^	689 (96.1)^a,b,c^	
Females	643 (17.3)^b,c,d,e^	174 (7.5)	258 (12.7)	39 (2.5)	28 (3.9)	
BMI, *n* (%)						0.209
non-obese	3,356 (90.5)	2,118 (91.4)	1,831 (90.1)	1,409 (92.0)	657 (91.6)	
Obese	352 (9.5)	200 (8.6)	202 (9.9)	122 (8.0)	60 (8.4)	
Hearing difficulty, *n* (%)						<0.001
No	2,914 (78.6)^c,d,e^	1,844 (79.6)^c,d,e^	1,555 (76.5)^a,b,e^	1,168 (76.3)^a,b,e^	474 (66.1)^a,b,c,d^	
Yes	794 (21.4)	474 (20.4)	478 (23.5)	363 (23.7)	243 (33.9)	
Tinnitus, *n* (%)						<0.001
No	2,600 (70.1)^c,d,e^	1,640 (70.8)^c,d,e^	1,370 (67.4)^a,b,d,e^	957 (62.5)^a,b,c,e^	404 (56.3)^a,b,c,d^	
Yes	1,108 (29.9)	678 (29.2)	663 (32.6)	574 (37.5)	313 (43.7)	
HPD use, *n* (%)						<0.001
<4 h/work-day	2,290 (61.8)^b,c,d,e^	1,719 (74.2)^a,d,e^	1,516 (74.6)^a,d,e^	1,256 (81.4)^a,b,c,e^	613 (85.5)^a,b,c,d^	
≥4 h/work-day	1,418 (38.2)	599 (25.8)	517 (25.4)	285 (18.6)	104 (14.5)	
Earphone use, *n* (%)						0.004
<1 h/day	2,151 (58.0)^c,d,e^	1,361 (58.7)^c,d,e^	1,112 (54.7)^a,b^	836 (54.6)^a,b^	384 (53.6)^a,b^	
≥1 h/day	1,557 (42.0)	957 (41.3)	921 (45.3)	695 (45.4)	333 (46.4)	
Tobacco consumption, *n* (%)						<0.001
<10 cigarettes/day	2,451 (66.1)^b,c,d,e^	1,371 (59.1)^a,c,d^	1,300 (63.9)^a,b,d,e^	886 (57.9)^a,c^	428 (59.7)^a,c^	
≥10 cigarettes/day	1,257 (33.9)	947 (40.9)	733 (36.1)	645 (42.1)	289 (40.3)	
Alcohol consumption, *n* (%)						<0.001
<50 g/day	3,023 (81.5)^b,c,d,e^	1,577 (68.0)^a,c,d^	1,498 (73.7)^a,b,d,e^	982 (64.1)^a,b,c,e^	478 (66.7)^a,c,d^	
≥50 g/day	685 (18.5)	741 (32.0)	535 (26.3)	549 (35.9)	239 (33.3)	

*SD, standard deviation; BMI, body mass index; CNE, cumulative noise exposure; HPD, hearing protective device*.

* *Comparisons were between different audiometric phenotypes*.

*Significantly different from normal hearing^a^, 4–6 kHz sharp-notched^b^, 4–6 kHz flat-notched^c^, 3–8 kHz notched^d^, and 1–8 kHz notched^e^ audiograms in post-hoc analyses of ANOVA or chi-square test (P < 0.05)*.

Variables that showed significant differences between audiometric phenotypes were included in the multinomial logistic regression analysis (Table 4). Age, male sex, tobacco consumption, and alcohol consumption were risk factors for all subtypes, while the HPD use was a protective factor. CNE was associated with three of all subtypes except for the 4–6 kHz flat-notched phenotype. Tinnitus was associated with three of all subtypes except for the 4–6 kHz sharp-notched phenotype. Self-reported hearing difficulty was only related to the 1–8 kHz notched phenotype, which reflected the most severe NIHL subtype.

**Table 4:** Multinomial logistic regression models of audiometric phenotypes.

**Variables**	**Refer to normal hearing**			
**(OR [95% CI])**	**4–6 kHz sharp-notched**	**4–6 kHz flat-notched**	**3–8 kHz notched**	**1–8 kHz notched**
Age (per years)	**1.09** (1.08–1.09)	**1.10** (1.09–1.10)	**1.16** (1.15–1.17)	**1.23** (1.22–1.25)
CNE (per dB[A])	**1.01** (1.01–1.01)	1.00 (1.00–1.01)	**1.02** (1.01–1.02)	**1.04** (1.03–1.05)
Male sex	**2.76** (2.27–3.35)	**1.72** (1.44–2.05)	**9.63** (6.81–13.63)	**6.44** (4.25–9.75)
Hearing difficulty (self-reported yes)	0.92 (0.80–1.06)	1.06 (0.92–1.22)	0.91 (0.77–1.08)	**1.36** (1.10–1.67)
Tinnitus (self-reported yes)	1.03 (0.91–1.17)	**1.19** (1.04–1.35)	**1.53** (1.32–1.78)	**1.84** (1.51–2.24)
HPD use ≥4 h/work-day	**0.84** (0.74–0.95)	**0.86** (0.75–0.98)	**0.77** (0.65–0.90)	**0.76** (0.59–0.97)
Earphone use ≥1 h/day	0.9 (0.81–1.01)	1.10 (0.97–1.23)	1.07 (0.93–1.22)	1.01 (0.84–1.22)
Tobacco consumption ≥10 cigarettes/day	**1.17** (1.03–1.32)	**1.10** (0.97–1.25)	**1.26** (1.09–1.45)	**1.33** (1.09–1.62)
Alcohol consumption ≥50 g/day	**1.51** (1.32–1.72)	**1.27** (1.11–1.47)	**1.54** (1.32–1.8)	**1.25** (1.02–1.54)

*Reference variables: Sex, females; Hearing difficulty, self-reported no; Tinnitus, self-reported no; HPD use, <4 h/work day; Earphone use, <1 h/day; Tobacco consumption, <10 cigarettes/day; Alcohol consumption, <50 g/day*.

Bold type: P < 0.05.

*OR, odds ratio; CI, confidence interval; BMI, body mass index; CNE, cumulative noise exposure; HPD, hearing protective device*.

### Specific Influence of Noise Exposure on Audiometric Phenotypes

To explore the specific influence of noise exposure dose on audiometric phenotypes among populations with different characteristics, we performed hierarchical regression analysis of audiometric phenotypes stratified by confounding factors (sex, age, CNE, HPD use, hearing difficulty, tinnitus, tobacco consumption, and alcohol consumption). According to the adjusted ORs of noise exposure dose for different phenotypes after stratification, the increment of CNE was stably associated with the 4–6 kHz sharp-notched phenotype (Figure 3B), as well as associated with the 3–8 kHz notched phenotype except among younger subjects (<30 years old) (Figure 5B) and the 1–8 kHz notched phenotypes except for females and younger population (Figure 6B). In contrast, CNE was almost unrelated to the 4–6 kHz flat-notched phenotype (Figure 4B), except for population who were males, with hearing difficulty and little tobacco consumption.

## Discussion

In this study we performed a cluster analysis of noise-exposed population who had some degree of hearing loss. By using the audiometric thresholds over 0.5–8 kHz of the total hearing loss dataset (*n* = 6,599), we developed the cluster model and identified four phenotypes with distinct audiogram subtypes of hearing loss. We repeated the cluster analysis in two independent parts of the total dataset, dataset 1 (*n* = 4,461) and dataset 2 (*n* = 2,138) where we were able to replicate the clusters into four similar phenotypes. The relevant demographic and behavioral characteristics of population with different hearing loss phenotypes were analyzed in comparison with the normal hearing population (*n* = 3,708).

Our main finding was that hearing loss in noise-exposed population consisted of four audiogram subtypes that had different characteristics and associations with noise exposure levels. In line with previous studies, we found the presence of a “notch” at high frequencies of 3, 4, and 6 kHz with recovery at 8 kHz in most hearing loss audiograms, some of which extended to involve even 1 kHz and 2 kHz (14–16). Therefore, we named the phenotypes 4–6 kHz sharp-notched, 4–6 kHz flat-notched, 3–8 kHz notched, and 1–8 kHz notched audiograms.

In the present study, the 4–6 kHz sharp-notched audiogram, 3–8 kHz notched audiogram, and 1–8 kHz notched audiogram were strongly related to noise exposure, which represented three distinct subtypes of NIHL. This result supported the conventional description of noise-induced high-frequency audiometric notches (8, 9, 23) based on data-driven evidence. The occurrence of 4–6 kHz sharp-notched audiogram was highest among all subtypes with constant correlation to the noise exposure, which could be regarded as a typical subtype of NIHL. While the 3–8 kHz notched audiogram and 1–8 kHz notched audiogram that manifested as more severe subtypes of NIHL involved wider ranges of frequency, which were less likely to appear among younger populations and even females. This finding agreed with several previous studies suggesting that the risk of NIHL in males was significantly higher than that in females (12, 15, 24), as well as the effects of aging may extend the hearing loss frequencies to 8 kHz and even low frequencies, which reduces the prominence of the typical “notch” in audiograms of individuals with excess noise exposure (8, 9).

In particular, the 4–6 kHz flat-notched audiogram was the second most common subtype of hearing loss after the 4–6 kHz sharp-notched audiogram, however, it seemed to be unrelated to noise exposure, but associated with age, sex, and some behavioral factors according to the logistic regression (Table 4). Although the mean CNE of subjects with the 4–6 kHz flat-notched audiogram was significantly larger than that of the normal-hearing audiogram, it might due to the longer working-length of subjects with the 4–6 kHz flat-notched audiogram, who were also older than those with the normal-hearing audiogram. In addition, the average hearing thresholds of the 4–6 kHz flat-notched audiogram at lower frequencies were higher than that of the 4–6 kHz sharp-notched audiogram, despite of the similar mean age, and CNE. However, in consideration of the obvious differences in sex, hearing difficulty, tinnitus, and earphone use between the two subtypes, we speculated that there should be other factors (such as individual behaviors and genetic heterogeneity) influencing the audiometric phenotypes, which should be further explored in future studies. This finding may provide an explanation for some previous studies reporting that audiometric notches also commonly occur in individuals without any previous noise exposure and have been associated with other factors (14, 15, 25). The Nord-Trøndelag Hearing Loss Study analyzed the various definitions of notched audiograms in the 3–6 kHz range [defined by Coles et al. (9), Hoffman et al. (26), Wilson and Mcardle (27)] in 49 774 subjects aged 20–101 years. The prevalence of those notches varied from 60 to 70% in the most noise-exposed men, but was also common in men without any occupational noise exposure. Another study using the Hoffmann notch to analyze audiograms of US adults from the NHANES (16) showed that though 8.2% of 1,223 self-reported occupational noise-exposed individuals had bilateral high-frequency audiometric notches, 5.2% of 2,360 individuals without noise exposure also had bilateral notches. Those artificial definitions of notches probably included this 4–6 kHz flat-notched audiogram, which may limit the specificity of using high-frequency audiometric notch for the diagnosis of NIHL.

As many previous studies reported (11, 28, 29), we found that age, sex, tobacco, and alcohol consumption were confounding influencing factors of hearing loss other than noise exposure. Using HPDs in an environment with loud noise exposure for hours every work-day likely protected individuals from NIHL, despite audiometric subtypes. In addition, we found that tinnitus was associated with the degree of hearing loss rather than the most typical NIHL subtype, while self-reported hearing difficulty was only closely related to the most severe subtype of hearing loss with speech frequencies impairment. These findings are approximately consistent with previous studies that reported that tinnitus is usually accompanied by hearing loss (30), and self-reported hearing status could not sensitively reflect high-frequency hearing loss (31).

It is widely accepted that audiometric phenotypes are based on presumed underlying auditory histopathology, which suggests the causes and degree of auditory organ damage (32, 33). A few previous studies have performed cluster analysis in clinical audiograms. Interestingly, the notched audiometric phenotype was always distinguished out as a separate cluster (18, 19), and we assumed that it should indicate the NIHL phenotype, although the noise exposure history of those patients was not reported. Here we propose to use this cluster classification to identify audiometric phenotypes for the evaluation of NIHL, since the typical NIHL in a specific population may manifest as different subtypes of notched audiograms, and suggest different management approaches. For instance, the presence of 4–6 kHz sharp-notched audiogram in younger females might be a strong signal indicating NIHL, in contrast, the 4–6 kHz flat-notched audiogram should not be evidence of NIHL. This would facilitate optimal assessment of NIHL.

The main strength of our study is that it first provides various reproducible audiometric subtypes of NIHL by data-driven analysis in a relatively large-scale noise-exposed population. Another strength was that our study was based on consideration of detailed noise exposure history, questionnaire information and audiometric data from standardized protocols, which can give a more nuanced picture than clinical data. Previously Zhao et al. developed machine learning models for the prediction of NIHL (34), which were based on hypothesis-driven or supervised analysis. Instead, for the first time to our knowledge, we performed an unsupervised data-driven cluster analysis to identify the unknown audiometric phenotypes associated with noise exposure, and to describe the relevant characteristics of distinct subtypes of NIHL. However, there are also some limitations. First, this cross-sectional study did not allow robust causal inference, although the employees were supposed to have a pre-work health examination to ensure normal hearing at baseline. Second, all subjects in this study were collected in the same region of China and they may not represent the whole noise-exposed population. Furthermore, we cannot at this stage claim that the new subtypes represent different etiologies of NIHL, or that this clustering is the optimal classification of NIHL phenotypes.

In conclusion, we were able to repeat and identify distinct audiometric phenotypes of NIHL in large-scale noise-exposed populations with different relevant characteristics, by using cluster analysis. Moreover, given the technological advances in machine learning, our study provides a sight into the prospect of involving data-driven audiogram mining for the precise diagnosis and treatment of NIHL in future studies.

## Data Availability Statement

The original contributions presented in the study are included in the article/supplementary material, further inquiries can be directed to the corresponding authors.

## Ethics Statement

The studies involving human participants were reviewed and approved by the Ethics Committee of the Ninth People's Hospital affiliated to Shanghai Jiao Tong University School of Medicine. The patients/participants provided their written informed consent to participate in this study.

## Author Contributions

QW, MQ, ZH, and HW: contributed to the study design. QW and MQ: writing the original draft. MQ, LY, JS, YH, and KH: contributed to the acquisition of data. QW, CL, and JL contributed to the analysis of data, ZH and HW contributed to the supervision and funding acquisition of the work, review and revision of the draft. All authors contributed to the interpretation of data and critical revision of the draft. All authors gave final approval of the version to be published.

## Conflict of Interest

The authors declare that the research was conducted in the absence of any commercial or financial relationships that could be construed as a potential conflict of interest.
